# No evidence for competition between cytotoxic T-lymphocyte responses in HIV-1 infection

**DOI:** 10.1098/rspb.2009.1232

**Published:** 2009-09-23

**Authors:** Helen R. Fryer, Almut Scherer, Annette Oxenius, Rodney Phillips, Angela R. McLean

**Affiliations:** 1 The Institute for Emerging Infections, The James Martin 21st Century School, University of Oxford, South Parks Road, Oxford, UK; 2 Department of Zoology, University of Oxford, South Parks Road, Oxford, UK; 3 ETH Zurich, Institute of Microbiology, Wolfgang-Pauli Strasse 10, 8093 Zürich, Switzerland; 4 The Peter Medawar Building for Pathogen Research, Nuffield Department of Clinical Medicine, University of Oxford, South Parks Road, Oxford, UK

**Keywords:** cytotoxic T-lymphocytes, human immunodeficiency virus, competition, mathematical model

## Abstract

Strong competition between cytotoxic T-lymphocytes (CTLs) specific for different epitopes in human immunodeficiency virus (HIV) infection would have important implications for the design of an HIV vaccine. To investigate evidence for this type of competition, we analysed CTL response data from 97 patients with chronic HIV infection who were frequently sampled for up to 96 weeks. For each sample, CTL responses directed against a range of known epitopes in *gag*, *pol* and *nef* were measured using an enzyme-linked immunospot assay. The Lotka–Volterra model of competition was used to predict patterns that would be expected from these data if competitive interactions materially affect CTL numbers. In this application, the model predicts that when hosts make responses to a larger number of epitopes, they would have diminished responses to each epitope and that if one epitope-specific response becomes dramatically smaller, others would increase in size to compensate; conversely if one response grows, others would shrink. Analysis of the experimental data reveals results that are wholly inconsistent with these predictions. In hosts who respond to more epitopes, the average epitope-specific response tends to be larger, not smaller. Furthermore, responses to different epitopes almost always increase in unison or decrease in unison. Our findings are therefore inconsistent with the hypothesis that there is competition between CTL responses directed against different epitopes in HIV infection. This suggests that vaccines that elicit broad responses would be favourable because they would direct a larger total response against the virus, in addition to being more robust to the effects of CTL escape.

## 1. Introduction

In human immunodeficiency virus-1 (HIV-1) infection, the cytotoxic T-lymphocyte (CTL) response is typically focused towards a handful of epitopes (Scherer *et al*. 2004). There is variation in the magnitude of the response to each epitope, and it has been argued that this apparent hierarchy is the result of competition between CTLs of different epitope specificities (Nowak *et al*. 1995). Whether there is competition between CTL populations directed against different epitopes has important implications for HIV vaccine design. Arguably, a vaccine that induces a broad CTL response would be favourable by directing a large total response against the virus and by being more robust if escape occurs. If, however, competition largely shapes the anti-HIV CTL repertoire, then a broader response may not be desirable because ineffective responses could suppress effective responses.

As yet, research into competition in HIV-1 infection is limited, though competition between CTLs has been shown to occur in mice in response to infection with herpes simplex virus-1 (Stock *et al*. 2006), influenza virus (Chen *et al*. 2000; Andreansky *et al*. 2005) and lymphocytic chroriomeningitis virus (Butz & Bevan 1998). Kedl *et al*. (2003) presented a review of the extensive body of research into T-cell competition in mice. They highlighted that competition can occur between CTLs specific for the same epitope (epitope-specific or interspecific competition) (Butz & Bevan 1998; Sandberg *et al*. 1998; Wolpert *et al*. 1998; Grufman *et al*. 1999*a*,*b*; Kedl *et al*. 2000, 2002; Smith *et al*. 2000; Probst *et al*. 2002) and between CTLs specific for different epitopes (intraspecific competition) (Sandberg *et al*. 1998; Wolpert *et al*. 1998; Grufman *et al*. 1999*b*; Chen *et al*. 2000; Kedl *et al*. 2000), though evidence for intraspecific competition is less compelling. In one experiment involving adoptive transfer of T-cells, it was shown that intraspecific competition is far less efficient than interspecific competition (Kedl *et al*. 2002) and in another there was no evidence of intraspecific competition at all (Probst *et al*. 2002). Similarly, deletion of dominant viral or bacterial epitopes has failed to significantly enhance responses directed against previously subdominant epitopes (Moskophidis & Zinkernagel 1995; Weidt *et al*. 1998), and in one such experiment, responses were not enhanced at all (Vijh *et al*. 1999).

In cases where competition has been observed in mice—both to the same and to different epitopes—antigen-presenting cells (APCs) have been shown to play a crucial role (Kedl *et al*. 2003). These cells present epitopes to naive T-cells, resulting in activation and proliferation of the T-cells specific to the epitopes presented. In epitope-specific competition, CTLs appear to downmodulate the level of the epitope presented on the APC after interaction with the APC (Kedl *et al*. 2002). This loss of antigens inhibits activation of other T-cells with the same specificity, particularly those with lower affinity for the epitope. Competition may also occur for access to antigens and costimulatory molecules at the APC surface. In intraspecific competition, downmodulation does not feature; instead it is thought that competition results solely from limited access to the surface of APCs (Wolpert *et al*. 1998; Borghans *et al*. 1999; Grufman *et al*. 1999*b*). The role of APCs in intraspecific competition is confirmed by the observation that intraspecific competition is abolished when different epitopes are presented on different APCs (Kedl *et al*. 2000).

With regard to competition between CTLs in HIV-1, one report by Nowak *et al*. (Nowak *et al*. 1995) presented data from two humans that demonstrate fluctuations in the CTL response directed against different epitopes and against different variants—i.e. mutant and wild-type—of the same epitope. In some cases, a decrease in one response coincided with an increase in another response, but whether these dynamics were driven by competition, rather than simply changes in the circulating virus strains, was unclear (Nowak *et al*. 1995). In a more comprehensive study (Altfeld *et al*. 2006), it was shown that the response to epitopes restricted by HLAs A1, A2 and A3 was lower in humans who also expressed HLA B57 or HLA B27 compared with those who did not express HLA B57 or HLA B27. The inference was that strong responses to the immunodominant epitopes TSTLQEQIGW (B57-restricted) and KRWIILGLNK (B27-restricted) compete with and suppress responses to other epitopes. Evidence for intraspecific competition also comes from mice immunized with CTL epitopes from different clades of HIV-1 (Larke *et al*. 2007). During single-clade immunization, a potent response was observed to the clade B epitope AMQMLK**E**TI, but this was ablated when the mice were simultaneously immunized with the clade A version of this epitope, AMQMLK**D**TI.

Detecting competition in whole organism ecology is a well-known, thorny problem. It can be very difficult to manipulate the abundance of individual species or groups of species in a natural setting (Dybzinski & Tilman 2009). Even if such manipulations are successful, it can be hard to interpret experimental outcomes. For example, if removing one species leads to no change in the density of its putative competitors is that because they are (and always have been) genuinely independent of each other, or is it that the species assemblage has grown up to minimize competitive interactions (Connell 1980)? Even if experimental removal of one species demonstrably leads to increases in the abundance of its competitors, questions remain about the mechanisms of competition. Were they competing for shared resources (Tilman 1982) or did they instead share predators at a higher trophic level (Holt 1977)? Careful combinations of mathematical models, observational data and experimental manipulations have led, over the past several decades, to much richer interpretations of the processes that drive the assembly of species into communities (reviewed in Keddy 2001; Tilman 2007). Although that theory was developed to explain whole organism ecology, much of it could be borrowed or adapted to explore the diversity of immune responses within individuals (McLean *et al*. 1997).

In this study, we investigate evidence for competition between CTL responses specific for different epitopes in HIV-1 infection by analysing CTL responses made by 97 chronically infected HIV-1 patients who were enrolled onto an intermittent therapy trial. Patients were frequently sampled for up to 96 weeks. For each sample, CTL responses directed against a range of known epitopes were measured using an enzyme-linked immunospot (ELISPOT) assay. To test for competition, we compared these data with the results predicted by the Lotka–Volterra model of competition. Specifically, we asked the following two questions:

(i) If many responses are present, is each response smaller?
(ii) If one epitope-specific response becomes dramatically smaller, do others increase in size in apparent compensation, and conversely if one response grows, do others shrink?

## 2. Material and methods

### (a) Patient cohort and study design

Ninety seven patients from Switzerland were recruited onto the Swiss–Spanish Intermittent Therapy Trial (SSITT), a large study devised to assess the clinical, virological and immunological outcome of structured treatment interruptions in individuals with chronic HIV infection. These individuals all gave informed consent before participating in the study and have been described and studied in detail elsewhere (Oxenius *et al*. 2002*b*; Scherer *et al*. 2004; Frater *et al*. 2007). Patients were only included in the study if their CD4 count was above 300 cells per mm^3^ at the time of enrolment and if they had been on continuous antiretroviral therapy (ART) with a plasma viral load less than 50 copies per ml for at least six months. The treatment interruption schedule required that ART was stopped at week 0 for two weeks, and resumed thereafter for eight weeks (weeks 2–10). This cycle was then repeated four times. Patients resumed continuous therapy and were removed from the study if their viral load persisted above 50 copies per ml during any of the periods on therapy. Patients were also removed from the study at week 40 if their CD4 count was less than 400 cells per mm^3^. For all other patients, therapy was stopped at week 40 for up to 56 weeks, i.e. until week 96, and was only resumed if symptoms associated with acute infection arose, or if viral load exceeded 500 000 copies per ml once, 100 000 copies per ml twice or 50 000 ml three times. While patients remained on the study, they provided blood samples at frequent intervals. The mean number of times that patients were sampled was 11 (range 1–24) and the mean period across which these samples were taken was 58 weeks (range 0–96 weeks).

### (b) Plasma viral load, viral subtyping and HLA typing

Plasma viral load was quantified throughout the study from cryopreserved plasma by using the regular or the ultrasensitive (for measurements taken on ART) Roche HIV Monitor assay (Roche Diagnostics, Rotkreuz, Switzerland; limit of detection 200 and 50 copies per ml). Viral loads fluctuated with the treatment cycles (Oxenius *et al*. 2002*b*), but remained comparatively stable beyond week 50, 10 weeks after the final cessation of therapy. As part of a study into immune escape mutants (Frater *et al*. 2007), viral DNA was extracted from peripheral blood mononuclear cells (PBMCs) collected while the patients were off therapy. HIV-1 *gag*, *pol* and *nef* genes were amplified by nested PCR and sequenced using ABI Big Dye terminator sequencing kits. Sequences were available from 88 of the Swiss patients, and the Rega Subtyping Tool (http://dbpartners.stanford.edu/RegaSubtyping/) was used to subtype these individuals. Seventy six per cent of the patients were found to be clade B. For each individual, human leukocyte antigen (HLA) class I genotypes were also determined by PCR using sequence-specific primers.

### (c) IFN-γ enzyme-linked immunospot assay

Throughout the study, CTL responses directed against peptides representing clade B optimal epitopes were measured *ex vivo* by gamma interferon production using an interferon-gamma (IFN-γ) ELISPOT assay (Oxenius *et al*. 2002*b*; Scherer *et al*. 2004). All but three of the epitopes tested had sequences equal to the HXB2 reference strain and responses were measured in terms of the number of spot-forming cells (SFC) per million PBMCs above the background level. According to HLA type, patients were screened against a median of 17 (range 0–31) peptides; across all HLA types, 80 peptides were tested. A description of the epitopes and the full set of data used in our analysis are presented in spreadsheet S1 in the electronic supplementary material. In addition, an example of the CTL response data available for a single individual is presented in table S1 in the electronic supplementary material.

### (d) Analysis of cytotoxic T-lymphocyte response data

To understand how the mean CTL response per epitope correlates with the number of epitopes targeted, the CTL response data was first analysed from a cross-sectional perspective. For each patient, at each time point, the number of screened epitopes that the patient made a response to was compared with the mean response per epitope. Only responses greater than 50 spot-forming cells (SFCs)/10^6^ PBMCs were regarded as positive responses in this analysis. For example, patient 72 was tested at 12 different time points, recording between 0 and 4 CTL responses at each time point. For each time point tested, this patient contributed a single data point relating the number of responses to the mean response size. Raw data for this patient (and all others) are given in electronic supplementary material, spreadsheet S1, and details of this cross-sectional data treatment are given in table S1*b* in the electronic supplementary material. Minitab statistical package was used to fit a linear correlation between these two variables by weighted least-squares regression analysis. Using Minitab, we also calculated the Pearson correlation coefficient (PCC) and the *p*-value relating to a two-sided hypothesis test in which the null hypothesis is that the PCC is zero.

To understand how large changes in the response to specific epitopes correlate with changes in the total response directed against all other epitopes, the SSITT data were also analysed longitudinally. Since changes in the CTL response could occur in response to changes in viral load caused by therapy interruption, only data from week 50 and beyond were analysed for this part of the study. By this stage patients would have been off therapy for 10 weeks, therefore fluctuations in CTL responses caused by therapy interruptions should no longer occur. Indeed, beyond this point, there was no evidence of systematic increases in either the viral load, the breadth of CTL responses or the magnitude of CTL responses over time. For each epitope, the difference in the response between available sample times for each combination of two sample times was calculated and the largest difference (in magnitude) observed in a single epitope was determined. The corresponding total change in the responses directed against all other screened epitopes at that time was also determined and the relationship between these two variables was calculated and tested using Minitab, as described above. For example, patient 72 was screened at four time points after week 50, yielding six possible longitudinal comparisons. The largest difference in response to a single epitope was seen in the response to DTGHSNQVSQNY that rose from 26 SFCs/10^6^ PBMCs at week 56 to 2843 at week 72 (an increase of 2817 SFCs/10^6^ PBMC). Over the same period, the sum of the responses to all other epitopes rose from 815 to 4292 SFCs/10^6^ PBMC (an increase of 3477 SFCs/10^6^ PBMCs), hence it is these two vectors (+2817 and +3477) that represent the longitudinal data from this patient (raw data in spreadsheet S1 and this analysis in the electronic supplementary material, table S1*c*). For completeness, we performed the same analysis on data from the whole time period that patients were studied.

### (e) Lotka–Volterra model of competition

To understand what patterns we would expect to see in the SSITT ELISPOT data under competition, we have investigated the dynamics of the Lotka–Voltera competition model (Volterra 1926; Lotka 1932; Murray 1989). For certain parameter sets, this model represents a system where *n* distinct populations can coexist in the setting of competition between both elements of the same population and elements of different populations. Here, we have used this model to represent competition between different populations of CTLs specific for different epitopes. The model can be expressed as a set of ordinary differential equations (equation (2.1)).2.1
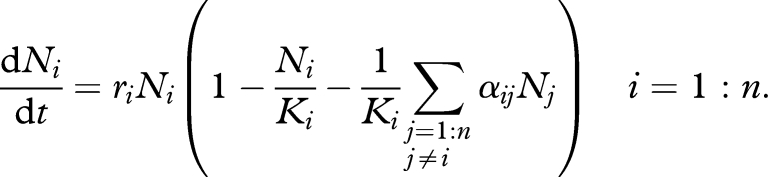

*N*_*i*_ refers to all the CTLs specific to one epitope (epitope *i*), which, from here on we will call a specificity. Population growth is density dependent, and in the absence of competition, the *per capita* growth rate of each CTL population is *r*_*i*_. For each specificity, growth is regulated by the carrying capacity, *K*_*i*_. This means that when all CTL populations specific to other epitopes are absent, the population size of specificity *i* would increase if its size (*N*_*i*_) were below the carrying capacity and decrease if its size were above the carrying capacity. In real terms, the carrying capacity for CTLs specific for a particular epitope represents a global measure of the presence of the components necessary for inducing an immune response to that particular epitope. This may include the abundance of the epitope presented on the surface of infected cells and/or APCs and the affinity that CTLs have for the epitope. In the model, competition between CTLs of different specificities can occur for those components required by all CTLs, such as the basic level of antigens presented. The competition coefficient, *α*_*ij*_, represents the effect that the CTL population of specificity *j* has on that of specificity *i*. If *α*_*ij*_ = 1, then the survival of CTLs of specificity *i* is affected equally by the presence of CTLs of specificity *j* as by the presence of other CTLs of specificity *i*, whereas if *α*_*ij*_ = 0, then the two specificities act independently. Different populations can coexist provided the effect of interspecific competition on each population is less important than the effect of intraspecific competition. In the simplest case of this model, where there are CTLs specific for two epitopes (*n* = 2), coexistence is possible provided *K*_1_ > *α*_12_*K*_2_ and *K*_2_ > *α*_21_*K*_1_, that is, provided *α*_12_ and *α*_21_ are sufficiently small in comparison with the carrying capacities, *K*_1_ *K*_2_.

**Figure 1.**
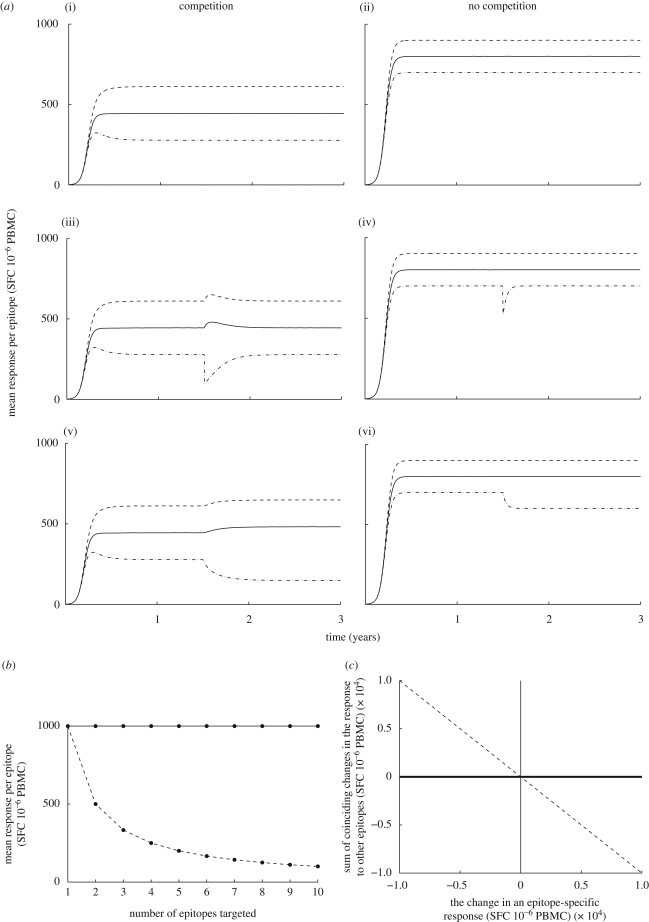
Model predictions. (*a*) Predicted behaviour under the Lotka–Volterra model in the presence and absence of competition between HIV-specifc CTL specificities. In (i), (iii) and (v), there is competition between CTLs specific for different epitopes, whereas in (ii), (iv) and (vi), there is no competition (*α*_*ij*_ = 0 for all *i* and all *j, i* ≠ *j*). In (i) and (ii) there is no change in CTL specific for either of three epitopes. In (iii) and (iv) there is a sporadic decrease in CTL specific for one of three epitopes (epitope 3). In (v) and (vi) there is a decrease in the carrying capacity of CTL specific for epitope 3. The figures show that if a decrease (or increase) occurs in the response to one epitope, then other responses will increase (or decrease) if there is competition between specificities. Changes in other responses will be temporary following a sporadic change in the response to one epitope, but permanent following a change in the carrying capacity of one epitope. The following model parameters were used: *K*_1_ = 900, *K*_2_ = 800 and *K*_3_ = 700; *r*_*i*_ = 30 for all *i* and *α*_*ij*_ = 0.4 for all *i* and all *j* when there is competition. The starting values were *N*_*i*_(0) = 0 for all *i*. In (iii) and (iv), the response to epitope 3 decreases by 200 at time *t* = 1.5 years. In (v) and (vi), the carrying capacity for CTLs directed against epitope 3 decreases to 600 at *t* = 1.5 years. Dashed lines, epitope 1; solid lines, epitope 2; dashed-dotted lines, epitope 3. (*b*) Model prediction of how the mean response per epitope varies with the number of epitopes targeted. If there is no interspecific competition (solid line), the mean response does not vary with the number of epitopes targeted, whereas if there is interspecific competition (dashed line) (*α*_*ij*_ > 0 for some *i* and *j*), the mean response per epitope decreases with the number of epitopes targeted. For this example, we have assumed that the carrying capacity, *K*_*i*_, of each epitope-specific response is the same (*K*_*i*_ = 1000) and that interspecific competition between each specificity is as strong as intraspecific competition (*α*_*ij*_ = 1 for all *i* and *j*). In this particular case, the mean response is inversely proportional to the number of epitopes targeted. (*c*) Model prediction of how the change in the response directed against a single epitope would correspond to the change in the sum of the responses directed against other epitopes. If there is no interspecific competition, significant changes in the response directed against one epitope would not correspond to changes in the total response directed against other epitopes (solid line). If there is interspecific competition, an increase in the response directed against one epitope would correspond to a decrease in the total response directed against other epitopes (dashed line). These changes would be exactly equal in magnitude (as shown) if interspecific competition is as strong as intraspecific competition.

For our analysis, we use this model to represent the scenario where coexistence of CTL populations specific to several epitopes is possible, since in HIV infection CTL responses are typically maintained against several epitopes within each host (Scherer *et al*. 2004). Figure 1*a* demonstrates the basic dynamics of three CTL populations of different specificities, as prescribed by this model. If the number of CTLs specific for each epitope is initially low, each population increases before settling at an equilibrium, which is determined by the degree of competition between the different CTL populations and the carrying capacities of the different specificities (figure 1*a*(i)(ii)). These equilibria are locally stable for most parameter values; therefore, if a sporadic decrease (or increase) occurs in the number of CTLs specific for one epitope, after a period of time the equilibria will be regained. In the interim, there will be a *temporary* increase (or decrease) in CTLs directed against other epitopes if competition occurs (figure 1*a*(iii)), but no change if no competition occurs (figure 1*a*(iv)). A second way in which the response directed against one epitope could change is if the carrying capacity of the epitope decreases (or increases). Rather than representing a sporadic change, this would represent a change in the ability of that particular CTL population to thrive irrespective of competition. An example might be a reduction in antigens bearing the wild-type epitope after outgrowth of an escape mutant. As above, if there is competition, then the long-term decline of CTLs of one specificity would coincide with the long-term growth of CTLs of other specificities. However, in this case the distinct CTL populations would settle at different (stable) equilibria (figure 1*a*(v)). If there is no competition, other CTL populations will not be affected (figure 1*a*(vi)).

## 3. Results

### (a) If many responses are present, is each response smaller?

To investigate whether there is competition between CTL responses directed against different epitopes, we first compared actual measurements with model predictions as to how the mean response per epitope correlates with the number of epitopes targeted.

Using the Lotka–Volterra model, we compared scenarios in which different numbers of CTL populations, each with the same carrying capacity, coexist. To compare these scenarios under competition we assumed that the degree of interspecific competition is the same regardless of the number of epitopes targeted and the same for each competitive interaction (*α*_*ij*_ = *α* for all *i* and *j*, except *i* = *j*). Figure 1*b* shows that the mean response per epitope would decrease as the number of epitopes targeted increases (dashed line). If, however, there is no interspecific competition (*α*_*ij*_ = 0 for all *i* and *j*, except *i* = *j*), then the mean response per epitope would be independent of the number of epitopes targeted (solid line).

**Figure 2.**
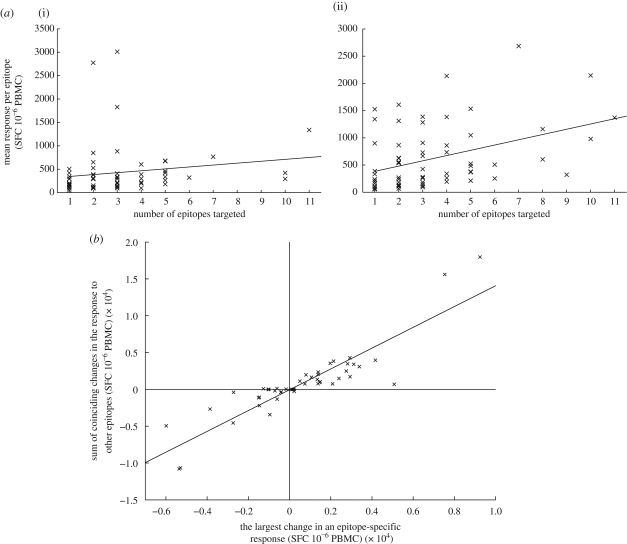
Observations from the SSITT data. (*a*) Patients with more responses make larger responses. A scatter plot and fitted line showing that there is a positive correlation between the mean response per epitope and the number of epitopes targeted for samples taken at (i) week 0 (not significant; PCC = 0.167, *p* = 0.191) and (ii) week 19 (significant; PCC = 0.405, *p* = 0.001), the two weeks with the most number of patients sampled (week 0, *n* = 73 and week 19, *n* = 69). When the same analysis was performed on the response data from each of the 29 different sampling times, a positive correlation was found at 26 sample times. Twelve of the positive correlations and none of the negative correlations were significant. The full analysis is presented in figure S1 in the electronic supplementary material. (*b*) A large change in one epitope-specific response is accompanied by changes in the same direction in other responses. A scatter plot and fitted line showing how the largest change in response directed against a single epitope corresponds to changes in the response directed against other epitopes. There is a striking positive correlation between these two variables (PCC = 0.897, *p* < 0.001), i.e. responses to different epitopes increase in unison or decrease in unison. Only changes corresponding to week 50 and beyond were included in this analysis. The data from each patient are represented by a single cross. The same pattern is observed when data from all sample times are analysed (PCC = 0.690, *p* < 0.001; data not shown).

We analysed the SSITT data to see how the mean response per epitope correlates with the number of screened epitopes targeted. This analysis was separately performed on data collected at each sample time and it was found that at the majority (90%) of sample times the mean response per epitope increased with the number of epitopes targeted (figure 2*a* of the main text and figure S1 in the electronic supplementary material), the opposite of what is predicted by the Lotka–Volterra model. At the remaining sample times, the mean response decreased as the number of epitopes targeted increased, though in no case was the negative correlation statistically significant. Taken together, these data therefore provide no evidence that competition exists between CTLs specific for different epitopes. Rather, they suggest that some overriding external factor drives the magnitude and breadth of the CTL response to increase or decrease in unison, or that CTLs are cooperative.

### (b) If one epitope-specific response becomes dramatically smaller, do others increase in size to compensate, and conversely if one response grows, do others shrink?

Next we investigated whether the within-host *dynamics* of CTL responses suggest that there is competition between responses. We compared data and model predictions of how large changes in one epitope-specific response correlate with changes in the responses directed against other epitopes. We used the Lotka–Volterra model to represent the dynamics of different coexisting CTL populations following a change in the carrying capacity of one epitope-specific CTL population. A change in carrying capacity drives a change (in the same direction) in the number of CTLs specific for that epitope. If there is competition between this epitope-specific CTL population and others, then an increase in the response to this epitope would result in a decrease in the total response directed against all other epitopes. Similarly, a decrease in the response to this epitope would result in an increase in the total response directed against other epitopes (figure 1*c*; dashed line, negative correlation). If, by contrast, there is no interspecific competition, then a change in the response to one epitope would not affect the response to any other epitopes (figure 1*c*; solid line, no correlation).

We analysed the CTL response data from week 50 and beyond to find how large changes in the response to particular epitopes correspond to changes in the response to all other epitopes. To perform this analysis, for each patient we calculated two variables: the largest change in the response to any epitope between any two time points and the change in the total response to all other epitopes over the same period. Figure 2*b* shows that there is a very strong positive correlation between these two variables (PCC = 0.897; *p* < 0.001), the opposite of what would be expected in a setting of interspecific competition. These findings were repeated when data from all time points were analysed (PCC = 0.690; *p* < 0.001; data not shown). The dynamical interpretation of the SSITT data is therefore also inconsistent with the existence of interspecific competition and instead reveals that expansions or contractions in different CTL populations occur in unison, either in response to other influential factors or through cooperation.

In summary, two patterns found in the CTL response data are inconsistent with predictions made by the Lotka–Volterra model under the assumption of competition between CTLs specific for different epitopes. Firstly, the mean response per epitope increases as the breadth of the response increases and, secondly, CTL responses against different epitopes tend to increase in unison or decrease in unison. Our very simple models of competition make no assumptions about what CTLs might be competing for. A different approach is to explicitly define that CTLs are competing for viral antigens. It is natural to ask whether models that include competition for antigens and acknowledge the antigenic diversity of HIV and corresponding effects on viral dynamics could explain the patterns we see. Certainly, it seems plausible that two or more competing CTLs could simultaneously fluctuate if antigens specific for those particular CTLs simultaneously fluctuate in the same direction. This could occur if the total level of all antigens fluctuates or if there are more isolated simultaneous fluctuations in the specific antigens considered. A model by Nowak *et al*. (1995) includes antigenic diversity and competition by CTLs for antigens and shows that the combination of these two factors could lead to complicated oscillatory dynamics of different viral strains and different CTL specificities. Among these dynamics, there are large fluctuations in the total abundance of virus, with the peaks being largely dominated by specific viral variants. Furthermore, competing CTLs targeting epitope variants that are linked on particular viral strains *can* simultaneously fluctuate in coincidence with fluctuations in those viral strains. By association, simultaneous fluctuations in CTLs also tend to coincide with changes in the total viral abundance. Finally, according to the Nowak model, patterns of simultaneous fluctuations of CTLs targeting linked epitope variants would become less marked with increased cross reactivity and with an increase in ‘mixed’ strains caused by recombination. So how do these issues affect how we should interpret the patterns that we have observed in our patients? To understand whether the *total* viral abundance is linked to the patterns we see, we have investigated how CTL responses compare to plasma viral load. We have found that changes in total viral abundance do not explain the patterns that we have observed. The breadth of the CTL response does not correlate with the log viral load (electronic supplementary material, figure S2*a*; e.g. PCC = −0.144, *p* = 0.261 at week 0 and PCC = 0.074, *p* = 0.699 at week 64) and large changes in epitope-specific responses do not correlate with changes in log viral load (electronic supplementary material, figure S2*b*; PCC = 0.065, *p* = 0.731). This still leaves the question of whether changes in the abundance of *specific* viral variants (but not the total viral abundance) could cause the patterns we see. We are not able to address this question directly because the extensive sequence data that would be required were not collected from our patients. From an indirect perspective, however, changes in the abundance of specific viral variants is not a valid explanation because we tend to see simultaneous fluctuations in the response to most, if not all, targeted epitopes within each individual (data not shown). Taken together, inferences from simple and complex models therefore suggest that our data provide no evidence in favour of competition between CTLs specific for different epitopes.

Our findings are contradictory to data presented by Altfeld *et al*. (2006), showing that hosts who express HLA B27 or HLA B57—alleles that induce well-described immunodominant responses—make lower responses to other epitopes. We repeated their analysis on our data, but we were unable to reproduce their results. In the presence of HLA B27/B57, responses to epitopes restricted by HLAs A1, A2 and A3 were no lower than in the absence of HLA B27/B57 (electronic supplementary material, figure S3).

## 4. Discussion

In this study, we have investigated evidence for competition between CTLs specific for different epitopes in HIV-1 infection. Analysis of CTL response data from a large cohort of chronically infected patients has revealed that this type of competition does not materially affect CTL numbers. We compared CTL response data with model predictions of how the mean response per epitope should correlate with the breadth of the response and how large changes in the response to one epitope should correlate with changes in the response to other epitopes. We found that the mean response per epitope increases as the breadth of the response increases and that large expansions in the response to one epitope correlate with expansions in the response to other epitopes. Both of these results are in contrast to the predictions made using the Lotka–Volterra model under the assumption of competition between CTLs specific for different epitopes. Our data are therefore inconsistent with the hypothesis of competition between HIV-specific CTLs. Instead they imply that CTL populations directed against different epitopes are cooperative or that external variables are the overriding influence on CTL numbers and affect different CTL populations in the same way. Antigenic stimulus is an obvious candidate for such an external variable; however, detailed analysis of the viral load data does not support this suggestion, adding weight to the assertion that CTLs specific for different epitopes do not compete.

Although these data show no signature that competition is acting between subspecificities of the HIV-specific response, there is clear evidence that at the level of the whole population—whether B cells (McLean *et al*. 1997) or T-cells (Veiga-Fernandes *et al*. 2000)—lymphocytes are subject to competitive interactions. Previous studies into competition between CTLs specific for different epitopes in mice have also revealed mixed findings. Some are in agreement with ours and have shown intraspecific competition to be absent or insignificant (Moskophidis & Zinkernagel 1995; Weidt *et al*. 1998; Kedl *et al*. 2002; Probst *et al*. 2002), whereas others have revealed evidence in favour of intraspecific competition (Sandberg *et al*. 1998; Wolpert *et al*. 1998; Grufman *et al*. 1999*b*; Chen *et al*. 2000; Kedl *et al*. 2000). Recent modelling work (Scherer *et al*. 2006) suggests that these differences could stem from the level at which epitopes are expressed on APCs in these different studies. Scherer and colleagues found that at low per APC expression levels CTLs compete for access to their cognate epitope, so there is competition between CTLs specific for the same epitope but not between CTLs specific for different epitopes. At high epitope expression levels, however, competition becomes aspecific, namely for access to the surface of the APC, resulting in intraspecific competition.

Our findings have important implications for HIV vaccine design since in the absence of competition any manipulation that broadens the CTL response could be beneficial by providing a larger total response against the virus and by providing a response that is more robust in the event of CTL escape in one or more epitopes. Further studies into competition between CTLs in HIV in different scenarios are crucial. The study we have presented was performed on patients who had established infections and competition may, for example, be present or more important during the initial response to the virus during acute infection or just after vaccination. Other factors that need to be ruled out as being influential to our findings are the method for the measurement of CTL responses and the patient cohort. Firstly, we note that for future studies, measurement of absolute numbers of CTL in a given volume of blood may be more informative than the ‘prevalence’ measure used here of the number of SFCs in a given number of PBMCs. Crucially though, a prevalence measure is intrinsically biased towards detecting a signature of competition even when none exists, meaning that our inference of no competition holds despite the counting method. Secondly, we note that the ELISPOT technique used here to measure interferon-γ has failed to explain differences between the magnitude of CTL responses and their effect on viral clearance rates (Oxenius *et al*. 2002*a*) and therefore may not reflect the true efficacy of the response. More recent studies indicate that the number of polyfunctional CTLs correlate with plasma viral load (Betts *et al*. 2006; Daucher *et al*. 2008), and it would therefore be interesting to investigate whether lack of competition is also observed when polyfunctional CTLs are measured. Thirdly, we acknowledge that many CTL epitopes to which patients make responses (wild-type and mutant variants) have not been screened in this study. Nevertheless, our ELISPOT dataset is one of the largest and most comprehensive to date and the patterns that we have observed are very clear. It is therefore likely that more comprehensive studies would reveal the same patterns. Finally, we note that different patient cohorts should also be investigated. The SSITT patients used in this study were unusual in that they had all undergone a series of structured treatment interruptions, though it is noteworthy that our findings were robust across the full period that the patients were studied. One particular set of individuals that would be interesting to investigate are patients enrolled onto post-infection cell-mediated therapeutic HIV vaccine trials. In our studies, we have used an indirect measure of competition, whereas the gold standard in studies of competition in whole organism ecology is experimental manipulations that add or subtract species. Although, at first sight such experiments seem unethical in humans, post-infection vaccine efficacy studies can be thought of as experimental manipulations in which CTLs of certain specificities are experimentally boosted. CTL responses pre- and post-vaccination are closely monitored in HIV vaccine trials and data from vaccinees would therefore be ideal for testing our prediction that CTLs specific for different epitopes do not compete.
